# Beyond the Solvent: Engineering Ionic Liquids for Biomedical Applications—Advances, Challenges, and Future Directions

**DOI:** 10.3390/molecules31020305

**Published:** 2026-01-15

**Authors:** Amal A. M. Elgharbawy, Najihah Mohd Noor, Nor Azrini Nadiha Azmi, Beauty Suestining Diyah Dewanti

**Affiliations:** 1 International Institute for Halal Research and Training, International Islamic University Malaysia, Jln. Gombak, Kuala Lumpur 53100, Malaysia; 2 Department of Agroindustrial Technology, Faculty of Agricultural Technology, Universitas Brawijaya, Jl. Veteran, Malang 65145, Indonesia; beauty_dewanti@ub.ac.id; 3 Kulliyyah of Engineering, International Islamic University Malaysia, Kuala Lumpur 50728, Malaysia; 4 Halal Products Research Institute, Universiti Putra Malaysia (UPM Serdang), Serdang 43400, Malaysia; azriniazmi@gmail.com

**Keywords:** ionic liquids, drug delivery, medical imaging, biosensors, lab-on-a-chip, biomedical applications

## Abstract

Ionic liquids (ILs) have emerged as multifunctional compounds with low volatility, high thermal stability, and tunable solvation capabilities, making them highly promising for biomedical applications. First explored in the late 1990s and early 2000s for enhancing the thermal stability of enzymes, antimicrobial agents, and controlled release systems, ILs have since gained significant attention in drug delivery, antimicrobial treatments, medical imaging, and biosensing. This review examines the diverse functions of ILs in contemporary therapeutics and diagnostics, highlighting their transformative capabilities in improving drug solubility, bioavailability, transdermal permeability, and pathogen inactivation. In drug delivery, ILs improve solubility of bioactive compounds, with several IL formulations achieving substantial solubility enhancements for poorly soluble drugs. Bio-ILs, in particular, show promise in enhancing drug delivery systems, such as improving transdermal permeability. ILs also exhibit significant antimicrobial and antiviral activity, offering new avenues for combating resistant pathogens. Despite their broad potential, challenges such as cytotoxicity, long-term metabolic effects, and the stability of ILs in physiological conditions persist. While much research has focused on their physicochemical properties, biological activity and in vivo studies are still underexplored. The future directions for ILs in biomedical applications include the development of bioengineered ILs and hybrid ILs, combining functional components like nanoparticles and polymers to create multifunctional materials. These ILs, derived from renewable resources, show great promise in personalized medicine and clinical applications. Further research is necessary to evaluate their pharmacokinetics, biodistribution, and long-term safety to fully realize their biomedical potential. This study emphasizes the potential of ILs to transform therapeutic and diagnostic technologies by highlighting present shortcomings and offering pathways for clinical translation, while also debating the need for continuous research to fully utilize their biomedical capabilities.

## 1. Introduction

Ionic liquids (ILs) are salt-like substances consisting of organic or inorganic cations, such as imidazolium or pyridinium, paired with anions including nitrate, acetate, tetrafluoroborate, dicyanamide, bis (trifluoromethyl sulfonyl) imide, and lactate. These substances remain in a liquid state at or below 100 °C [1]. Beyond their role as solvents, ILs have attracted considerable scientific attention due to their unique physicochemical properties, including low volatility, high thermal stability, tunable solubility, and potential biocompatibility. These characteristics make ILs promising candidates for a wide range of applications in modern therapeutics and diagnostics, such as drug delivery, tissue engineering, biosensing, and regenerative medicine. In contrast to simple salts like sodium chloride (NaCl), which contain small and symmetrical ions (Na^+^ and Cl^−^) that are efficiently packed into a highly ordered crystalline lattice, the ions that make up ILs are typically much larger, more asymmetrical, and often have flexible side chains or delocalized charges. These structural features hinder close packing and further reduce lattice energy, preventing the formation of stable crystalline structures. Thus, ILs remain liquids under ambient conditions [2].

Ethylammonium nitrate (C_2_H_8_N_2_O_3_), the first known room-temperature molten salt and the first example of a protic ionic liquid (PIL) that had a melting point of 12 °C, was the first IL discovered by Paul Walden in 1914 [3]. The lower melting point of an IL can be attributed to the bulky and unsymmetrical structure of the constituent ions, which can be paired together in different combinations to tailor their thermophysical properties [4]. Moreover, the charge on ions in ILs is distributed over a larger volume of molecules by resonance; thus, the solidification of ILs occurs at lower temperatures [5]. Appearing around the beginning of the 1960s, the first generation of ILs was fascinating due to their unique and adjustable physical features, for example, density, viscosity, thermal stability, low volatility, vast liquid ranges, and conductivity. This generation, however, had downsides, mostly because they were sensitive to exposure to air and water. Consequently, the second generation of ILs emerged in the 1990s as a response to the growing demand for solvents with tunable physical and chemical characteristics [3]. By employing independently altered cations and anions, such task-specific ILs were produced, hence facilitating the development of new functional materials. The advancements in second-generation ILs have enabled enhancements in chemical reactivity, chiral recognition, solvation, oxygen balance, UV blocking, and high energy density. Additionally, this category of ILs exhibited greater stability and a more positive environmental profile, as they were derived from biodegradable ionic sources. The scientific interest in ILs also developed in the 1990s, and scientists started systematically naming and classifying these new compounds. Ionic liquids are salts composed entirely of ions that exhibit melting points below 100 °C, often remaining liquid at or near room temperature. New ILs with specific characteristics were synthesized, opening up a wide range of potential uses. During the 2000s, research on ILs expanded rapidly, resulting in the identification of numerous applications. Simultaneously, the recently emerged principles of green chemistry demanded researchers focus on renewable raw materials, reduce waste, and avoid the use of toxic and hazardous reagent solvents [6].

**Figure 1:**
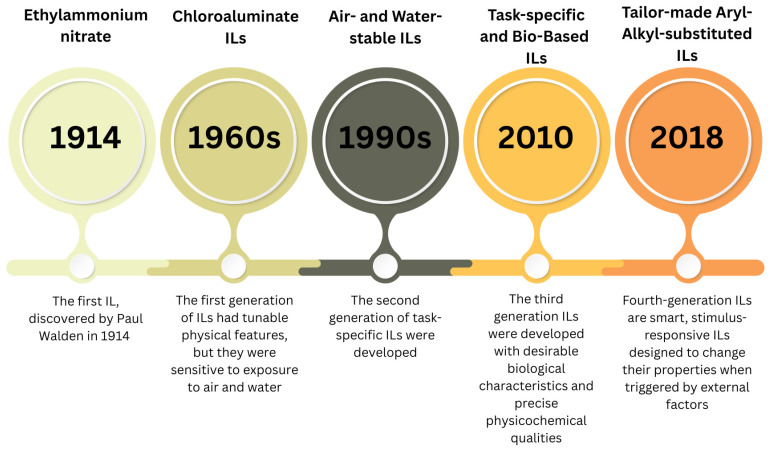
The timeline development of ILs from the first to fourth generation.

In line with these green chemistry goals, ILs emerged as suitable alternatives to conventional volatile solvents. ILs were identified as superior solvents for a diverse array of chemical and inorganic substances. They were employed in catalysis, separation processes, electrochemistry, and as lubricants. The unique adjustability of their characteristics via molecular design has created new opportunities for scientific investigation and industrial use. The research on ILs in the 2010s significantly highlighted their potential as environmentally friendly and sustainable solvents. Their minimal volatility, non-flammable nature, and recyclability render them appealing substitutes for conventional organic solvents, many of which are hazardous to the environment [7]. The beginning of the 21st century marks the emergence of the third generation of ILs [8], which merge desirable biological characteristics with precise physicochemical qualities. This group includes occurrences of ILs utilized in medicine, such as active pharmaceutical precursors or ingredients (APIs). Cations with proven low toxicity and advantageous effects (antibacterial, antifungal, anticholinergic, and local anesthetic) and anions possessing specific attributes (emollients, vitamins, antibiotics [9], and NSAIDs) have been employed to achieve the desired biological features of the ILs [10]. In 2018, a new class of imidazolium-based ionic liquids, known as tunable aryl-alkyl ionic liquids (TAAILs), emerged as a promising alternative to conventional ILs. These ILs contain a substituted aryl ring attached to one of the imidazole nitrogen atoms, enabling precise tuning of steric and electronic properties through variations in the aryl substitution pattern. Their enhanced ability to dissolve metal salts makes TAAILs particularly useful for metal extraction and various catalytic applications [11]. The timeline of the emergence of ILs throughout the years is illustrated in Figure 1.

Customized ILs have numerous biological applications due to their unique ability to solubilize various molecules and adapt to different uses. They have been investigated as drug delivery systems [8,12,13] due to their strong ionic conductivity and capacity to solvate a diverse array of molecules, allowing the controlled release of active substances over prolonged periods. The proper selection of ions during formulation promotes the capacity of the IL to improve drug solubility, while also enabling the synthesis of targeted biological functions based on the selected mode of administration [14]. ILs were first used in biomedical nanocarriers to synthesize and template silica nanoparticles for antibacterial, gene transfection, and medication release systems [15]. ILs have also been studied for use in tissue engineering and wound healing [16,17], either as solvents for biomaterials or as agents that enhance cell proliferation and tissue regeneration. For example, 1-butyl-3-methylimidazolium chloride ([BMIM][Cl]) and 2-hydroxyethyl-trimethylammonium dihydrogen phosphate ([Ch][DHP]) have been incorporated into poly(vinylidene fluoride) (PVDF) composites, improving electrical conductivity, inducing polar β-phase crystallization, and supporting C_2_C_12_ myoblast proliferation, demonstrating their potential as electromechanically active scaffolds for muscle tissue engineering [18]. Their antimicrobial properties [19] have also led to their use in medical coatings, offering potential as biocompatible materials for implants and prosthetics that reduce the risk of infection [20]. The hydrophobic alkyl chain component of the IL penetrates the bacterial cell membrane, leading to membrane disruption and, ultimately, cell death. The antibacterial efficacy is also associated with the hydrophobic characteristics of an IL [19]. Furthermore, ILs have been studied in the context of diagnostics, where their ability to stabilize proteins and enzymes has led to their incorporation in diagnostic devices and biosensors. Other than that, IL-based biosensors have displayed their effectiveness in monitoring and detecting biomolecules within the pharmaceutical field, demonstrating excellent thermal stability, minimal volatility, and comparatively wide electrochemical stability window under controlled conditions. It should also be noted that the usable electrochemical window of ILs is highly dependent on the electrode material, operating temperature, and the presence of trace water or protic impurities, and may differ significantly between neat IL systems and IL–aqueous or buffer-based mixtures [21]. This is proven by their ability to detect an extensive array of biomolecules, including glucose, hormones, nucleic acids, and pivotal biomarkers. Their electrochemical properties allow them to immobilize the biological analytes on the surface of the electrode, allowing this new generation of biosensing devices to deliver very precise measurements with extremely low detection limits [8].

In the last three years, bio-ionic liquids have evolved from “green solvents” to functional excipients and API-ILs, improving solubility, permeability, and bioavailability for challenging drugs while supporting enzyme stabilization and biopharmaceutical processing. Parallel innovations in molecular imaging—such as zero-background ^19^F MRI—demonstrate IL-based or fluorinated probes with enhanced sensitivity and multiplexing, complementing conventional ^1^H imaging. Despite these advances, thorough assessment of toxicity, pharmacokinetics, biodistribution, and long-term safety remains essential to ensure clinical translation. This review uniquely emphasizes biological evaluation and clinical relevance, identifies critical gaps in mechanistic understanding, and explores innovative strategies, including bioengineered and hybrid ILs for personalized medicine [22]. By addressing these underexplored aspects, we aim to provide guidance for future research that advances the safe and effective use of ILs in biomedical applications.

## 2. Therapeutic Applications of Ionic Liquids

In recent years, the pharmaceutical industry has increasingly explored the industrial-scale application of ILs to tackle persistent issues such as polymorphism, low solubility, poor permeability, instability, and the limited bioavailability of crystalline drugs [23]. ILs have advanced beyond their role as traditional solvents, offering tailored solutions for specific therapeutic needs. Their ability to enhance the solubility of poorly soluble or insoluble drugs and improve drug permeation has proven essential for developing new formulations and increasing the therapeutic efficacy of existing drugs [24].

### 2.1. Drug Delivery Systems

Advancements in drug delivery systems (DDSs) have significantly improved the precision and efficiency of delivering therapeutic agents. Despite these developments, numerous challenges still limit their effectiveness. These include variability and inconsistency in research data, poor absorption and bioavailability, and instability of delivery systems. Biological barriers, such as enzymatic degradation and selective permeability of the blood–brain barrier (BBB), further complicate the delivery of drugs to specific target sites [25].

ILs, with their tunable properties, have emerged as promising candidates to address these challenges in DDSs. In drug delivery, ILs have been used in several distinct ways: as neat solvents to dissolve poorly soluble drugs, as formulation additives that increase the apparent solubility or dissolution of drugs in aqueous systems, and as active pharmaceutical ingredient ionic liquids (API-ILs), where the drug itself is converted into IL form through ion pairing. ILs have also been incorporated as permeation enhancers, stabilizing agents, polymerization media, and functional components of nanocarriers to improve drug transport, stability, and bioavailability [26].

Table 1 provides a conceptual summary highlighting the various applications of ILs in DDSs, focusing on their roles in enhancing drug solubility and improving bioavailability.

Mechanistic insights from the IL-drug systems listed in Table 1 demonstrate how ILs enhance drug solubility, bioavailability, and targeted delivery through distinct physicochemical interactions. For instance, choline-based ILs paired with amino acids such as [Ch][Gly] significantly increased the aqueous solubility of ACV from less than 0.5 mg/mL in water to approximately 250 mg/mL in [Ch][Gly]. Even the least effective IL, [Ch][Ser], achieved a solubility of ~135 mg/mL, reflecting a >500-fold enhancement. This was attributed to multiple molecular interactions, particularly hydrogen bonding, van der Waals forces, or π–π interactions between the IL and ACV [36], which disrupted the crystalline lattice and prevented aggregation. The glycinate anion’s high surface electron density enabled stable complex formation [37], while its moderate viscosity supported efficient drug dissolution without significantly hindering diffusion, thereby contributing to its solubilising ability. For edaravone, combination with tetrabutylphosphonium cations increased solubility from 1.96 mg/mL to 3.47 mg/mL and led to the spontaneous formation of negatively charged nanoparticles (~199 nm) upon aqueous dispersion [30]. These nanoparticles, which exceeded the glomerular filtration threshold, evaded rapid renal clearance and prolonged systemic circulation. Additionally, they reduced renal accumulation, potentially lowering nephrotoxicity. In targeted delivery, ciprofloxacin was incorporated into IL-functionalized mesoporous silica nanoparticles (IL-MSNs), enabling site-specific delivery through electrostatic interactions [34]. ILs based on cholinium and 1-methylimidazolium provided a positive surface charge, promoting electrostatic interaction with the negatively charged bacterial membranes of *Klebsiella pneumoniae*, resulting in a 10-fold reduction in minimum inhibitory concentration compared to free ciprofloxacin. The non-crystalline encapsulation also allowed sustained, controlled release, enhancing antimicrobial efficacy while minimizing off-target effects.

While several IL-drug systems demonstrate promising improvements in solubility and in vitro performance, the extent of pharmacokinetics and toxicity benefits remains limited to the available data. For edaravone, the IL formulation produced nanoparticles that showed reduced renal toxicity and altered circulation behavior compared to free edaravone. In IL-MSNs, ciproflaxin exhibited improved antimicrobial activity at lower doses, which may reduce overall drug exposure. For poorly soluble drugs such as acyclovir, [Ch][Gly] ILs increased solubility over 500-fold, eliminating the need for organic co-solvents or surfactants associated with irritation or toxicity. These outcomes highlight the potential of ILs to enhance drug performance while reducing reliance of excipients that may introduce adverse effects.

Taken together, these examples show the versatility of ILs in pharmaceutical design. By tailoring cation–anion combinations, ILs can be optimized to form stable complexes or functionalized delivery systems that improve solubility and bioavailability.

The effectiveness of IL formulations is also demonstrated in their disease-specific applications. For example, in cancer treatment, ILs such as [EDMPC][Linoleate] have been shown to improve the transcutaneous delivery of immunomodulators [38]. This strategy boosts immune responses, including increased IgG and CD8+ T-cell activity, resulting in more effective tumor suppression compared to conventional formulations. Similarly, for neurodegenerative conditions, Wu et al. [39] developed IL formulations of donepezil to improve its delivery for Alzheimer’s treatment. By combining donepezil with anions like docosahexaenoic acid (DHA) and α-linoleic acid, the ILs showed up to 1.9 times better skin permeability than regular donepezil. These ILs were used in transdermal patches, which released the drug more effectively and had better mobility due to lower glass transition temperatures. The ILs also crossed the BBB well and retained strong acetylcholinesterase (AChE) inhibition, with DHA-based ILs being the most effective. Some ILs, such as docusate-based ones, were less toxic than standard donepezil, making them safer and more promising for treating neurodegenerative diseases. In a study by Nurunnabi et al. [40], an IL known as choline and geranate (CAGE) was developed to reduce fat absorption in the intestine, thereby addressing obesity-related diabetes. CAGE formed micelles with dietary fats, preventing their absorption and reducing weight gain in rats by 12% over 30 days. It also decreased food intake, likely by promoting satiety. Safety tests showed no adverse effects on organs or blood markers. The adaptability of ILs thus positions them as a promising solution for developing safer and more effective treatments across a broad spectrum of diseases that benefit from targeted, improved delivery methods.

### 2.2. Antimicrobial and Antiviral Properties

Bacteria naturally employ various mechanisms to evade the action of antibiotics, allowing them to survive treatments that were once effective. As antimicrobial resistance increases worldwide, it has become a critical public health concern associated with increasing morbidity and mortality. The widespread use of antibiotics provides bacteria with more opportunities to develop resistance, rendering many antibiotics ineffective against certain pathogens. This often necessitates higher doses or the use of alternative antibiotic classes [41,42,43].

**Figure 2:**
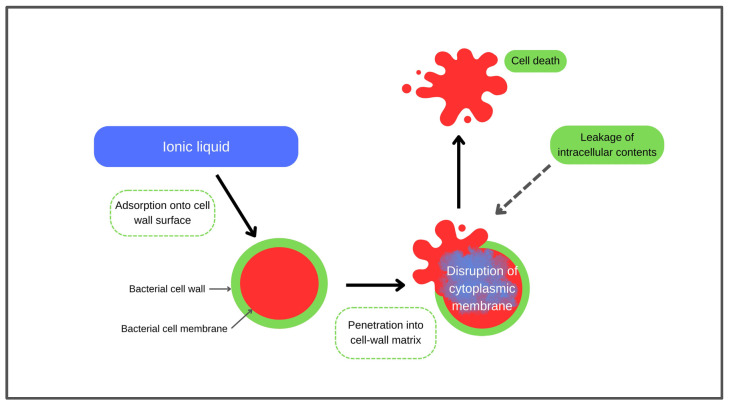
Schematic of the antimicrobial mechanism of ILs acting on bacterial cell membranes. The diagram illustrates the stages from electrostatic attraction of ILs to the bacterial membrane to membrane rupture, leakage of intracellular contents, and cell death. Solid arrows indicate direct interactions and primary mechanistic steps, while dashed arrows represent secondary or consequential processes following membrane disruption.

ILs have emerged as potential antimicrobial agents with mechanisms that differ from conventional antibiotics. Rather than targeting specific bacterial enzymes or pathways, ILs exert their antimicrobial activity through physicochemical disruption of the bacterial envelope [19]. The process begins with adsorption of cationic ILs onto negatively charged surface structures, including the cell wall, where electrostatic attraction can initiate structural weakening. Hydrophobic alkyl chains or non-polar regions of the ILs then penetrate the cell wall matrix, causing thinning or loss of structural integrity and exposing the cytoplasmic membrane [44]. Once bound to the membrane, ILs interact with phospholipids and membrane-associated proteins, leading to disturbed lipid packing, increased membrane fluidity, and impaired barrier functions. These effects hinder essential cellular processes such as molecular transport and nutrient uptake, ultimately leading to cell death. In some systems, pore formation has also been reported as part of the terminal damage pathway, contributing to irreversible cellular damage [19]. Figure 2 illustrates the antimicrobial mechanism of ILs.

In this context, the antimicrobial behavior of ILs is strongly influenced by structural features that govern lipophilicity and membrane affinity, including alkyl substitution patterns and overall molecular dimensions. While longer hydrophobic substituents generally enhance membrane interactions, molecular size and flexibility also play an important role in determining whether ILs penetrate bacterial membranes or primarily induce membrane destabilization [45,46]. Zheng et al. [47] reported the antibacterial properties of flexible ionic liquid derivatives (ILDs) with different molecular sizes (ranging from 1.95 to 4.2 nm) against Gram-negative bacteria such as *Escherichia coli* and *Pseudomonas aeruginosa*. The study showed that smaller ILDs, like ILD-6, are more effective in rapidly killing bacteria by penetrating their membranes, whereas larger ILDs, such as ILD-12, cause destabilization of the bacterial membrane without entering the cells. The size of the ILDs influences their interaction with bacterial membranes: smaller ILDs penetrate the membrane, while larger ILDs tend to insert into the lipid bilayer, weakening its structure.

Further study by Gundolf et al. [48] explores the effectiveness of antimicrobial active ILs against multidrug-resistant (MDR) bacteria, addressing the role of bacterial efflux pumps in IL resistance. The study demonstrates that ILs can interact with bacterial cells in multiple ways, including disrupting cell integrity, destabilizing proteins, and causing oxidative stress or DNA damage. The mechanism of IL action involves disrupting bacterial membranes and proteins, particularly in *Escherichia coli* and *Salmonella enterica* serovar Typhimurium. However, the presence of efflux pumps, such as the Resistance Nodulation-Division (RND) family, can reduce the efficacy of ILs. These pumps actively export antimicrobial agents from the bacterial cell, lowering their intracellular concentration. The study shows that ILs with one elongated alkyl side chain are more susceptible to efflux pumps, whereas ILs with multiple side chains demonstrate less susceptibility to pump-mediated resistance.

Similarly, imidazolium ILs have demonstrated strong antifungal activity, especially against *Candida albicans*, a common fungal pathogen. ILs with dodecyl and hexadecyl groups effectively inhibit fungal growth and biofilm formation, even in fluconazole-resistant strains [49]. The most potent IL, [C_16_MIM][Cl], not only prevents biofilm formation but also disperses pre-formed biofilms. The antifungal action of [C_16_MIM][Cl] is attributed to its ability to disrupt fungal cell membranes, increase membrane permeability, cause leakage of intracellular materials, reduce ergosterol content, and generate reactive oxygen species.

While the antimicrobial application of ILs has been extensively studied, their antiviral activity remains an emerging field of interest. Recent findings demonstrate that the antiviral efficacy of ILs is influenced by both the physicochemical properties of the ILs and the structural characteristics of the target virus. In a study by Faisca et al. [32], seven HCQ-ILs were synthesized by pairing the hydroxychloroquine cation with various anionic counterparts to improve solubility and antiviral efficacy. Among these, [HCQ][C_1_SO_3_]_2_ and [HCQH_2_][GlcHOO]_2_ demonstrated the most promising results, showing a two-fold reduction in cytopathic effect when compared to the parent salt, [HCQH_2_][SO_4_]. Although no statistically significant changes in intracellular viral RNA were observed, [HCQH_2_][GlcHOO]_2_ significantly reduced progeny virus production (*p* < 0.05), indicating interference at a post-replication stage of the viral lifecycle. Mechanistically, the antiviral activity of HCQ-ILs was not merely dependent on their hydrophilic or lipophilic balance, but likely due to specific intra- and intermolecular interactions, possibly involving viral envelop destabilization, inhibition of viral entry or fusion, or alterations in host endosomal pathways. These proposed mechanisms are consistent with the known mode of action of hydroxychloroquine, which interferes with endosomal acidification and receptor glycosylation [50,51], but may be enhanced via IL incorporation due to better membrane permeability and molecular flexibility. Importantly, both ILs exhibited no additional cytotoxicity at the effective concentrations, supporting their use as safer, structurally modified antiviral agents. In a recent study, Michalski et al. [52] evaluated the virucidal activity of 12 morpholinium-based ILs containing either a [Dec_2_Mor]^+^ or [DecEtMor]^+^ cation against five bacteriophages with different structural features: Phi6 (enveloped), P001 and P100 (non-enveloped tailed phages), and PRD1 and MS2 (non-enveloped icosahedral viruses). Among them, Phi6 was the most sensitive, with all [Dec_2_Mor]^+^ ILs inactivating the virus by $\geq 4\;{log}_{10}$ at 70–100 mg/L, while [DecEtMor]^+^ ILs required 1000–10,000 mg/L. The difference was linked to stronger membrane-disrupting ability of the more hydrophobic [Dec_2_Mor]^+^ cation, which contains two long decyl side chains. For non-enveloped viruses, P001 was inactivated by all ILs but required higher concentrations: 1000 mg/L for [Dec_2_Mor]^+^ and 10,000 mg/L for [DecEtMor]^+^. P100 was more resistant, with virucidal effects observed only for [Dec_2_Mor][Clopyralid] at 27,000 mg/L and [Dec_2_Mor][Dicamba] at 17,286 mg/L. No ILs were effective against PRD1 or MS2, even at 50,000 mg/L, highlighting their structural robustness. The study found that ILs act primarily by disrupting viral envelopes and interacting electrostatically with virus surfaces. The increased hydrophobicity [Dec_2_Mor]^+^ IL enhances their interaction with lipid membranes, while electrostatic binding may explain the inactivation of non-enveloped phages like P001. Structural differences, such as the presence of a contractile tail sheath in P100, were suggested to contribute to resistance by limiting access to vulnerable sites [53]. Overall, cation structure, particularly the number and length of alkyl chains, was the main determinant of virucidal activity, whereas anionic effects were generally minor and only noticeable in specific pairings such as clopyralid and dicamba.

Direct comparisons between ILs and established antiviral treatments are limited, and this remains a critical gap in the field. Future studies should aim to not only confirm the antiviral efficacy of ILs across various viruses but also establish standardized methodologies to compare their activity against conventional antiviral agents.

### 2.3. Anti-Inflammatory and Analgesic Uses

ILs also have been explored for their potential anti-inflammatory and analgesic properties. Chantereau et al. [54] developed NSAID-based ILs by pairing cholinium cations with ibuprofen, ketoprofen, and (S)-naproxen anions. These ILs exhibited water solubility up to 100 times higher than their NSAID counterparts, leading to improved bioavailability and easier incorporation into hydrophilic drug delivery systems. When incorporated into bacterial nanocellulose membranes, the ILs formed stable, biocompatible, and non-cytotoxical topical drug delivery systems with excellent rehydration and controlled drug release. In vitro studies demonstrated their effectiveness in reducing inflammation by lowering prostaglandin E2 production and LPS-induced nitric oxide levels, while avoiding side effects commonly associated with oral NSAIDs. Similarly, Bastos et al. [29] investigated ibuprofen-based ILs, including 1-ethyl-3-methylimidazolium ibuprofenate ([C_2_C_1_Im][Ibu]), 1-(2-hydroxyethyl)-3-methylimidazolium ibuprofenate [C_2_(OH)C_1_Im][Ibu], and cholinium ibuprofenate [N_1112_(OH)][Ibu]. These ILs not only retained the therapeutic benefits of ibuprofen but also demonstrated improved selectivity for cyclooxygenase-2 (COX-2), minimizing side effects associated with COX-1 inhibition, such as gastrointestinal discomfort. Their anti-inflammatory efficacy was validated through the inhibition of bovine serum albumin (BSA) denaturation and COX activity assays. In a recent study, Zhang et al. [55] developed an IL by combining ketoprofen (a NSAID) and actarit (a disease-modifying anti-rheumatic drug) with triethylamine as the counterion, yielding a dual-drug IL stabilized by semi-ionic hydrogen bonding. This IL was incorporated into a pressure-sensitive adhesive transdermal patch (SIHDD-PSA), which exhibited enhanced drug loading and skin permeability. Pharmacokinetic studies in Wistar rats demonstrated that the SIHDD-PSA patch provided sustained plasma concentrations of both drugs with improved bioavailability compared to conventional oral and commercial transdermal formulations. Furthermore, in a Complete Freund’s adjuvant (CFA)-induced rheumatoid arthritis rat model, the IL-based patch significantly reduced paw swelling, inflammation, and bone erosion. Histological and radiological assessment confirmed decreased synovial hyperplasia and joint destruction, while Western blot analysis showed modulation of the JAK-STAT signaling pathway. These in vivo results substantiate the therapeutic potential of NSAID-based ILs not only for improved drug delivery but also for effective management of chronic inflammatory diseases such as rheumatoid arthritis.

Beyond their anti-inflammatory benefits, ILs have also shown promise in enhancing analgesic effects. This is attributed to their ability to amplify the pharmacological properties of NSAIDs. For example, studies on novel 8-piperazinyl caffeine (8-PC) carboxylate ILs have shown synergistic activity when paired with NSAID anions such as ibuprofenate and naproxenate [56]. In vivo formalin tests confirmed that these ILs produced analgesic efficacy during the acute pain phase compared to their conventional sodium salt forms. The enhanced effect is attributed to the 8-PC cation’s interaction with the COX-2 enzyme, a key mediator of inflammatory pain. Molecular docking studies further validated this mechanism, showing strong binding affinity of the ILs to the COX-2 active site through hydrogen bonding, van der Waals forces, and electrostatic interactions. These findings highlight the mechanistic basis for the analgesic and anti-inflammatory performance of IL-based systems.

Building on this, ILs offer several advantages over traditional anti-inflammatory drugs in treating inflammation and pain. Their enhanced solubility, ability to selectively inhibit specific enzymes, and potential for reduced side effects make them an appealing option. The use of ILs in controlled-release formulations and synergy with NSAIDs further distinguish them as a promising class of compounds for targeted treatment of inflammatory diseases and pain management. Nonetheless, further studies are required to confirm their long-term safety and efficacy, but current evidence suggests that ILs may provide a more efficient and side-effect-reduced alternative to conventional NSAIDs.

## 3. Mechanistic and Biological Studies on Ionic Liquids

ILs are unique solvents whose properties are primarily determined by their specific combination of cations and anions. Computational techniques, such as Density Functional Theory (DFT), molecular dynamics, and COSMO-RS (Conductor-like Screening Model for Real Solvents) are often employed to explore the interactions between ILs and solutes at the molecular level [57,58,59]. These approaches provide insights into the solvation mechanism in ILs, which differs from those of conventional solvents due to the ionic nature of ILs. The distinct properties of ILs make them particularly attractive for biological applications. Their ionic composition enables them to interact with biological systems in unique ways, influencing factors such as solubility, stability, and bioactivity. These characteristics make IL valuable in a variety of biological applications, including antimicrobial and anticancer therapies, as well as DDS. However, the biological activity of ILs is organism-dependent, and their toxicity can vary depending on the specific structure of the IL [10]. Therefore, it is important to tailor the design of ILs to specific therapeutic targets rather than relying on a universal formulation. Such a “one size does not fit all” approach ensures improved efficacy, safety, and compliance in the use of ILs for biological applications.

### 3.1. Interaction with Biological Membranes and Cells

**Figure 3:**
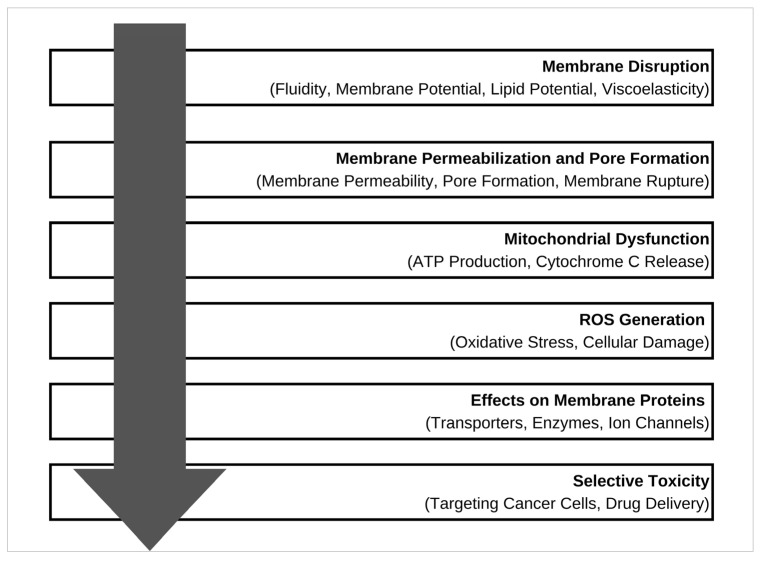
Mechanisms of ILs on biological membranes and cells, adapted from [60]. Published by Springer Nature. Licensed under Creative Commons Attribution 4.0 International (CC BY 4.0) (https://creativecommons.org/licenses/by/4.0/) [60].

The interaction of ILs with biological membranes and cellular components is a critical area of research, especially in understanding their biological behavior and potential applications. Figure 3 shows several mechanisms through which ILs interact with biological membranes and cells.

The mechanisms by which ILs interact with cellular structures vary depending on their concentration and specific chemical composition. At lower concentrations, ILs may cause reversible changes in cellular structures and functions, such as alterations in membrane fluidity or protein conformational changes [61]. These changes are often sub-lethal and do not immediately impair cellular activity. However, at higher concentrations, ILs can compromise membrane integrity, disrupt ion homeostasis, and interfere with enzymatic functions, leading to more severe outcomes. These include irreversible cellular damage through apoptosis and necrosis.

Huda et al. [62] demonstrated the therapeutic potential of choline octanoate (COA) as a carrier for the BCL-2 inhibitor navitoclax (NAVI) in treating early-stage skin melanoma. Their findings showed that COA increased NAVI’s solubility, improved skin penetration, and prolonged retention in dermal and subcutaneous layers. In vitro studies confirmed that the COA/NAVI combination induced apoptosis in melanoma cells, as shown by reduced viability and increased lactate dehydrogenase (LDH) release, indicative of cell membrane damage. Apoptosis was attributed to the inhibition of the BCL-2 protein, which plays a key anti-apoptotic role in cancer cells. In vivo, COA/NAVI reduced tumor volume more effectively than orally administered NAVI. Another study investigated the cytotoxic effects of 1-decyl-3-methylimidazolim chloride ([C_10_MIM]Cl) on HeLa cells [63]. At lower concentrations, [C_10_MIM]Cl caused mild oxidative stress and cell cycle arrest, effects that were potentially reversible, with cells recovering upon removal of the IL. However, higher concentrations led to irreversible damage, including membrane rupture, mitochondrial swelling, vacuolization, and apoptosis, ultimately resulting in cell death. The study linked these severe effects to oxidative damage, lipid peroxidation, and protein structure alterations, emphasizing that higher IL doses can cause permanent cellular damage.

Despite the promising properties of some ILs, such as low toxicity and high solubility in biological systems, others exhibit cytotoxicity, which limits their therapeutic application. Cytotoxicity is influenced by the composition of ILs, including cation and anion types, alkyl chain length, and overall hydrophobicity [64]. Delgado et al. [65] reported that ILs with longer alkyl chains and hydrophobic anions, such as bis(trifluoromethylsulfonyl)imide(Tf_2_N)^−^, exhibited higher toxicity, with pyridinium-based ILs being more toxic than imidazolium-based ones. Similarly, Kusumahastuti et al. [66] found that IL toxicity increased with alkyl chain length. Shorter chains (C2–C6) are less toxic, while longer chains (C10 and above) tend to be more toxic. However, for chains longer than C10, toxicity levels plateau or even decrease due to solubility issues or the formation of aggregates. Additionally, ILs with longer alkyl chain can accumulate in cell membranes, increasing their potential for absorption. However, very long chains may reduce bioavailability because they can form aggregates or have lower solubility.

### 3.2. Biocompatibility and Toxicology

Many traditional ILs can be toxic and not environmentally friendly. As the use of ILs in the biomedical field progresses, there is a clear shift toward designing ILs with reduced toxicity. The development of ILs from the first to the fourth generations reflects ongoing improvements in their structures, aimed at creating safer, more effective and sustainable solutions. The second and third generations are designed to be more compatible, making them safer for biological applications [67]. This generational transition not only focuses on modifying individual ions for reduced toxicity but also emphasizes biodegradability and biocompatibility to ensure safer interactions with biological systems.

Biocompatible ILs are typically synthesized from naturally available compounds and renewable sources [2]. Their design prioritizes low toxicity and environmental sustainability, with many being biodegradable and suitable for use in eco-friendly applications [68]. These ILs are often produced via metathesis or neutralization reactions, enabling precise tailoring of cation–anion combinations to tune physicochemical and biological properties. In line with this approach, Jopp et al. [69] developed a series of carbohydrate-based ILs (CHILs), particularly glucose-derived imidazolium ILs, as an alternative to conventional ILs known for their cytotoxicity and limited degradability. The biocompatibility of these CHILs was evaluated using L929 mouse fibroblasts, a standard model for assessing cytotoxic effects of materials in skin-contact applications. The results demonstrated that most CHILs showed high cell viability at concentrations of 10^−2^ mol/L, with viability generally exceeding 80–90% at this level, outperforming commercial imidazolium-based ILs in terms of cytocompatibility. Another study focused on the development of tetrabutylphosphonium (Tbp)-based ILs with medium-chain fatty acids as anions, specifically hexanoate, octanoate, decanoate, and dodecanoate [70]. The results showed varying levels of biocompatibility, with the fatty acid chain length affecting their biological activity. The ILs were more effective against Gram-positive bacteria, with longer fatty acid chains, particularly dodecanoate, showing the strongest antimicrobial effects. The ILs also exhibited good thermal stability, with their viscosity and conductivity changing based on the fatty acid chain length.

Novello et al. [45] reported imidazolium-based ILs with favorable biocompatibility and controlled cytotoxicity profiles, making them promising candidates for biomedical applications. In vitro cytotoxicity assays showed that ILs with shorter alkyl chains such as C_9_mimBF_4_ and C_9_mimDMSIP displayed limited cytotoxicity against MCF-7 breast cancer cells, with ${IC}_{50}$ values exceeding 14.81 μg/mL and 24.13 μg/mL, respectively, while still exerting moderate effects on HDF cells (${IC}_{50}$= 5.14 μg/mL and 4.96 μg/mL), respectively. In contrast, longer-chain ILs such C_16_mimBF_4_ and C_16_mimDMSIP exhibited strong antiproliferative activity against MCF-7 cells, with ${IC}_{50}$ values of 1.64 μg/mL (4.16 μM) and 3.84 μg/mL (6.61 μM), respectively. These ILs also affected HDF cells, but at higher ${IC}_{50}$ thresholds of 8.26 μg/mL (20.95 μM) and 5.56 μg/mL (9.57 μM), indicating an alkyl-chain-length-dependent change in cytotoxic response. Similarly, Xing et al. [71] developed a library of 61 ILs with systematically varied cationic side chains, cationic head groups, and anions, and observed that biocompatibility decreased progressively with increasing cationic alkyl chain length, whereas modifications to the cationic head group or anion contributed minimally to overall toxicity. Higher viability outcomes were reported for short-chain ILs across cell, spheroid, and patient-derived organoid models compared with long-chain counterparts.

Recent advances emphasize a design-for-safety approach, where toxicity is addressed at the molecular level through rational selection of cations, anions, and functional groups [72]. Strategies include shortening alkyl chain lengths, incorporating bio-derived ions (e.g., cholinium, amino acid, or carbohydrate-based moieties), and introducing biodegradable linkages to facilitate metabolic clearance [72,73,74]. Such bio-inspired and task-specific ionic liquids enable retention of functional performance while significantly reducing cytotoxicity and bioaccumulation risks, supporting their suitability for biomedical translation.

Collectively, these studies align with ongoing multidisciplinary efforts aimed at developing ILs that are not only functionally efficient but also exhibit reduced cytotoxicity and improved environmental compatibility. The ability to tailor biological responses through strategic structural modifications, incorporation of biocompatible or naturally derived components, and rigorous in vitro evaluation represents a significant advancement in the biomedical development of ILs. Continued interdisciplinary work in both molecular design and practical toxicological assessment will be essential in translating these next-generation ILs into safe, sustainable, and effective platforms for pharmaceutical and medical applications.

### 3.3. Metabolism and Clearance

Research into the metabolism and clearance of ILs within biological systems, particularly mammalian models, remains limited, leaving gaps in the understanding of how these unique compounds behave post-administration. Most available information on the fate of ILs comes from environmental biodegradation studies rather than detailed pharmacokinetic analyses in humans or animals.

Leitch et al. [75] investigated the biotransformation of 1-methyl-3-octylimidazolium (M8OI) using human hepatocytes and reported the formation of ω-hydroxylated metabolite (HO8IM) and a carboxylated metabolite (COOH7IM). The study also found that COOH7IM could replace lipoic acid in the mitochondrial pyruvate dehydrogenase complex (PDC-E2), suggesting that certain metabolites may bind to mitochondrial proteins instead of being cleared. However, no in vivo pharmacokinetic, biodistribution, or excretion data were reported, and therefore the clearance behavior of both the parent IL and its metabolites remains unknown. Similarly, Young et al. [76] examined long-term oral exposure to methylimidazolium-based ILs in mice and reported that M8OI was absorbed following oral administration, with both the parent compound and its metabolites (HO8IM and COOH7IM) detected in bile and urine, indicating both renal and biliary excretion. M8OI showed very low serum concentrations at termination, suggesting rapid systemic clearance and low bioavailability. The study also reported significant alterations in gut microbial composition in M8OI-exposed mice, while no marked liver or kidney injury was observed under the tested conditions.

Attention has also been directed towards understanding how ILs behave in environmental systems. Mena et al. [77] reported on the environmental impact of imidazolium- and choline-based ILs, focusing on biodegradability and toxicity using activated sludge to simulate wastewater treatment conditions. The study found that imidazolium-based ILs, particularly those with NTf_2_^−^ as an anion, were not biodegradable. On the other hand, choline-based ILs, such as [Ch][Cl] and [Ch][Ac], were biodegradable and degraded rapidly. The study also revealed that NTf_2_^−^ containing ILs were highly toxic and inhibited microbial activity in wastewater treatment processes. In contrast, ILs containing chloride or acetate anions were less toxic, especially those based on choline cations. Ecotoxicity data further confirmed that imidazolium-based ILs were generally more toxic than choline-based ones. The presence of NTf_2_^−^ increased the toxicity, making these ILs more harmful to the environment. The study provides valuable information on toxicity but does not directly address the metabolism of ILs by the liver, their excretion through the kidneys, or their enzymatic breakdown. More targeted research is needed to map out the metabolic pathways and clearance mechanism of ILs in biological systems. Such studies would greatly aid in the rational design of next-generation ILs with improved safety profiles. It is important that ILs not only remain effective in their intended applications but also ensure long-term safety for biological systems, preventing harm to the body over time.

## 4. ILs as Imaging Agents in Diagnostics

Diagnostic imaging modalities, such as magnetic resonance imaging (MRI), positron emission tomography (PET), and computed tomography (CT) scans, are essential in modern medicine for the identification and surveillance of diseases. These techniques generally depend on contrast agents to enhance image quality and diagnostic accuracy. ILs have emerged as viable alternatives or supplements to traditional imaging agents, owing to their distinctive physicochemical properties, including low volatility, high thermal stability, and adjustable solvation characteristics [78]. Moreover, their ability to solubilize various compounds and their biocompatibility render them appropriate candidates for augmenting imaging techniques, potentially resulting in enhanced diagnostic results.

### 4.1. Mechanisms and Functionality

Ionic liquids (ILs) have gained increasing attention in diagnostic imaging due to their high thermal stability, tunable physicochemical properties, and low volatility, which make them attractive functional media for imaging-related applications. Their role is currently most established in magnetic resonance imaging (MRI) and is increasingly emerging in positron emission tomography (PET) radiochemistry, particularly in the context of radionuclide stabilization and radiotracer synthesis.

In PET imaging, ILs have been investigated primarily as reaction media and stabilizing environments for radionuclides, rather than as imaging agents themselves. Fluorine-18 (^18^F), the most widely used PET radionuclide, is particularly sensitive to radiolytic degradation and solvent effects during synthesis and formulation. Several studies have demonstrated that ILs can promote radiolabeling reactions, simplify synthesis routes, reduce by-product formation, and shorten reaction times, thereby improving overall radiochemical efficiency [79]. These features are especially advantageous for ^18^F-labeling, where rapid and efficient synthesis is essential due to the short half-life of the radionuclide.

ILs also exhibit high solubility for metal ions and favorable buffering capacity, which contribute to improved stability and effectiveness of ^18^F-labeled radiotracers during preparation and handling [79]. In addition, understanding the radiation chemistry of ILs has revealed that their unique ionic environments can influence radiolysis pathways, providing insights into mitigating degradation mechanisms and preserving radiotracer integrity under irradiation [80,81]. Such properties are critical for maintaining radiochemical purity and imaging reliability in PET applications.

Importantly, the use of ILs has been shown to enhance radiochemical yield and stability of specific ^18^F-labeled tracers. For example, optimization of IL-assisted synthesis methods for [^18^F]FB-IL-2 resulted in a 3.8-fold increase in radiochemical yield compared with conventional approaches, while maintaining high radiochemical purity and stability [82]. These improvements directly translate into increased imaging efficiency and reduced material waste, both of which are important for clinical radiopharmaceutical production.

Beyond synthesis, IL-enabled ^18^F-radiotracers have been applied in biologically relevant PET imaging contexts. [^18^F]FB-IL-2 has been successfully employed for imaging activated T cells, providing valuable insights into immune activation and enabling assessment of responses to immunotherapies. In neurological applications, IL-facilitated radiotracers such as [^18^F]PBR146 have demonstrated utility in monitoring neuroinflammation, including in models of chronic hepatic encephalopathy, highlighting the potential of IL-based strategies to enhance brain imaging performance [83].

The stabilizing effects of ILs are further supported by their non-volatile and low-flammability characteristics, which reduce solvent loss during synthesis and storage while improving operational safety [79,82]. Moreover, the tunable nature of IL cation–anion combinations allows optimization of solubility, polarity, and interaction with radiolabeled compounds, offering a flexible platform for radiotracer formulation [82]. Integration of ILs into microfluidic radiochemistry systems has also been reported, enabling faster reaction kinetics, improved yields, and more controlled production of ^18^F-radiotracers suitable for clinical workflows [84,85].

Despite these promising developments, the application of ILs in PET imaging remains largely at the preclinical and radiochemistry development stage. Concerns regarding biodistribution, clearance, and potential bioaccumulation must be addressed through systematic pharmacokinetic and toxicological studies before broader clinical translation can be achieved. Nevertheless, current evidence supports the role of ILs as enabling materials in PET radiotracer synthesis and stabilization, rather than direct imaging agents, positioning them as valuable tools for advancing radiopharmaceutical science.

### 4.2. Applications

The studies reviewed focus on the use of amino acid-based paramagnetic ionic liquids (PMILs) as dual-mode (T1 and T2) MRI contrast agents, which offer enhanced imaging accuracy and biocompatibility. In a study conducted by Gehlot et al. [86], iron-containing amino acid-based PMILs with designated compositions, namely [Pro][FeCl_4_], [ProC_1_][FeCl_4_], [Glu][FeCl_4_], and [GluC_1_][FeCl_4_], were synthesized and evaluated. These PMILs exhibited enhanced MRI contrast characteristics relative to conventional gadolinium-based agents, demonstrating greater stability under physiological conditions and negligible DNA damage. The same PMILs ([Pro][FeCl_4_], [ProC_1_][FeCl_4_], [Glu][FeCl_4_], [GluC_1_][FeCl_4_], and [ValC_1_][FeCl_4_]) were studied by Gehlot & Kumar [87]. The relaxivity values and MRI performance of [AlaC_1_][FeCl_4_] were further characterized, showing dual T1 and T2 contrast effects that facilitated enhanced imaging with reduced contrast agent concentrations. The application of iron-based amino acid-derived PMILs (e.g., [Pro][FeCl_4_] and [Glu][FeCl_4_]) in MRI represents a significant improvement over traditional gadolinium-based agents, owing to their dual-mode imaging functionalities and improved biocompatibility. Nonetheless, clinical validation and scalability continue to pose significant obstacles to their extensive implementation.

**Figure 4:**
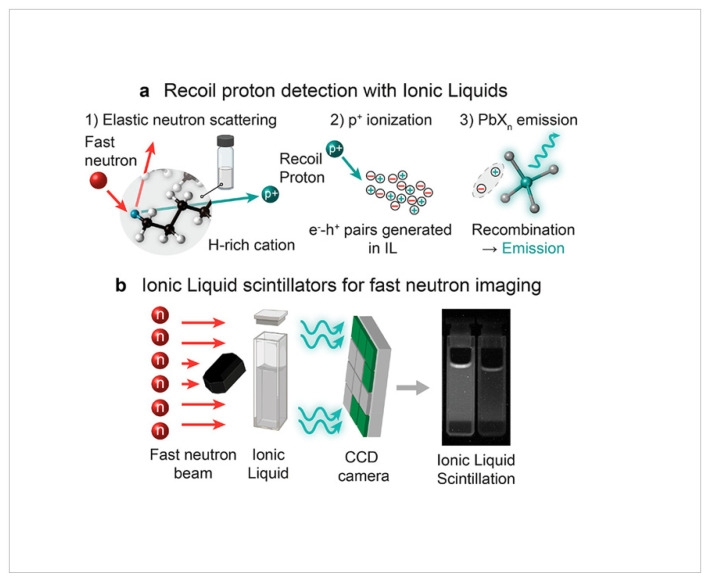
(**a**) Schematics of the processes involved in the recoil proton detection with ILs: (1) elastic neutron scattering on the H-rich cation with the generation of a recoil proton, (2) ionization of the recoil proton with subsequent generation of the electron–hole pairs in IL, and (3) recombination of the electron–hole pairs and emission of visible light photon. (**b**) Scheme of the indirect fast neutron imaging experiment with ILs as a scintillator. Reproduced from [89]. Published by the American Chemical Society. Licensed under CC-BY-NC-ND 4.0 (https://creativecommons.org/licenses/by-nc-nd/4.0/) [89].

Zhu et al. [88] formulated fluorinated ionic liquid (FIL)-based activatable ^19^F MRI probes for zero-background imaging, addressing the constraints of traditional ^1^H MRI. Encapsulated FILs emit an “off” signal until stimulated by particular biological triggers, resulting in a “turn-on” ^19^F signal for accurate imaging. This method provides elevated specificity devoid of background interference and improved diagnostic precision in biological systems (Figure 4).

Studies on 0D organic–inorganic Pb(II) halide ionic liquids have reported their application as fast neutron scintillators based on recoil proton detection. The materials consist of proton-rich organic cations and dense PbX_2_ emitting centers, forming room-temperature ionic liquids with large Stokes shifts (up to 1.7 eV), photoluminescence quantum yields up to 60%, and minimal light reabsorption. Optical characterization demonstrated efficient visible emission under fast neutron irradiation, while imaging experiments showed higher spatial resolution and lower γ-ray sensitivity compared with commercial ZnS:Cu-based scintillator screens [89]. The improved spatial resolution, reduced γ-ray sensitivity, and fast response of these scintillators enhance diagnostic neutron imaging by enabling clearer, more reliable visualization of internal structures in both medical applications, such as therapy verification and implant imaging, and industrial or defense settings involving dense or shielded objects.

Despite promising developments, significant challenges remain for IL-based imaging agents. Toxicity, bioaccumulation in soft tissues, and the stability of ionic liquids under physiological conditions pose notable limitations. For instance, bioaccumulation could lead to adverse long-term effects due to persistent IL residues in biological tissues, thereby compromising patient safety [90]. Moreover, ensuring consistent stability and performance of ILs during diagnostic procedures remains critical, requiring extensive validation under varied physiological environments [91]. Additionally, manufacturing scalability of IL-based contrast agents presents practical hurdles due to complex synthesis routes and purification requirements, potentially impacting clinical translation and commercial viability [92].

To mitigate these challenges, current research advocates the development of hybrid IL systems and biodegradable coatings. Hybrid IL systems combine the advantageous properties of ILs with biocompatible carriers or nanoparticles, reducing toxicity and bioaccumulation while enhancing their imaging efficacy and targeted delivery [13]. Additionally, the application of biodegradable coatings can significantly enhance IL stability and facilitate controlled degradation, reducing long-term bioaccumulation and potential toxicity [93]. These innovative approaches represent promising avenues toward safer, more effective, and clinically viable IL-based imaging agents.

## 5. Biosensors and Biomarkers

ILs have become essential elements in the progression of biosensor technology, especially in electrochemical and optical biosensors. In electrochemical biosensors, ILs function as electrolytes or additives to augment electron transfer rates, thereby enhancing sensor performance [94,95]. For example, IL-based glucose sensors exhibit remarkable sensitivity and specificity, facilitating real-time glucose monitoring in diabetic individuals. Likewise, ILs have demonstrated considerable promise in identifying cancer biomarkers, thereby aiding in the early diagnosis of malignancies through improved analyte interaction and signal resolution [94]. ILs were used to develop a highly sensitive and selective SPE-based electrochemical biosensor for non-invasive saliva detection of the HCC biomarker AFP, achieving an LOD of 0.058 ng/mL with a strong analytical performance. The sensor also demonstrated excellent environmental compatibility, reflected in an AGREE greenness score of 0.85, supporting its suitability for sustainable point-of-care diagnostics [96].

In optical biosensors, ILs function as stabilizers for fluorescent or colorimetric systems, enhancing both sensitivity and signal stability. Their minimal volatility and elevated thermal stability further augment the accuracy and dependability of biosensing applications. Table 2 below shows some examples of ILs used in biosensors.

The distinct roles of specific ILs has been highlighted, such as [C_4_mpyr][NTf_2_], [BMIM][BF4] and [BMIM][NTf_2_], in boosting sensor performance through enhanced biomolecule stability, improved electron transfer efficiency, and reduced biofouling. ILs have been effectively combined with advanced nanomaterials such as multiwalled carbon nanotubes (MWCNTs), graphene oxide (GO), titanium dioxide (TiO_2_), and gold nanoparticles (AuNPs), leading to enhanced sensitivity, selectivity, and stability in biosensors. The applications range from the detection of cancer biomarkers such as PSA, CD44, and AFP to wearable diagnostic platforms that monitor cytokines like IL-6 and stress hormones such as cortisol. The adaptability of ILs is showcased in non-invasive diagnostic tools, including saliva and sweat-based sensors, as well as in conventional electrochemical configurations utilizing phosphate-buffered saline (PBS) and serum samples.

IL-based biosensors demonstrate consistently low detection limits, wide detection ranges, and excellent reproducibility, rendering them ideal for point-of-care diagnostics and real-time health monitoring. As the field advances, the combination of ILs with sustainable and environmentally friendly designs, particularly those that align with the AGREE metric, underscores an increasing emphasis on eco-conscious sensor development. ILs are vital in advanced biosensing platforms, providing scalable, dependable, and exceptionally sensitive solutions for biomedical and healthcare applications.

## 6. In Vitro Diagnostics and Lab-on-a-Chip Systems

The incorporation of ILs into in vitro diagnostics and lab-on-a-chip (LOC) systems has become a ground-breaking method in analytical chemistry and biomedical applications. This literature review brings together current research on the use of ILs in microfluidic devices, highlighting their distinctive properties and the benefits they provide in diagnostics and sensor technologies.

ILs are known for their minimal vapor pressure, significant thermal stability, and adjustable properties, which render them suitable for a range of applications in microfluidics and diagnostics [102]. Their distinct physicochemical characteristics, including elevated ionic conductivity and the capacity to dissolve a broad spectrum of compounds, enable the creation of sophisticated microfluidic systems that can execute intricate analyses using minimal sample volumes [103]. The low volatility of ILs effectively tackles a major issue in microfluidic systems, where solvent evaporation can result in inconsistent outcomes and diminished reliability [104,105].

Numerous studies have demonstrated the application of ILs in LOC systems, highlighting their ability to improve the performance of microfluidic devices. For example, Yoon et al. documented the integration of ILs into microfluidic strain sensors, allowing the devices to preserve both transparency and functionality when subjected to mechanical stress [106]. This clarity is essential for the optical detection techniques frequently employed in diagnostics. Moreover, the capacity to manipulate ILs within microfluidic channels enables precise control over fluid dynamics, thereby improving the efficiency of assays and sensor applications [107,108].

ILs function as efficient solvents in the creation of nanomaterials in microfluidic systems. Yao et al. illustrated the application of ILs for the continuous synthesis of CeO_2_ nanostructures, emphasizing the benefits of microfluidic methods in obtaining consistent particle sizes and shapes [109]. This feature is especially advantageous for biosensing applications, where surface area and reactivity play crucial roles [110]. Furthermore, the integration of ILs into microfluidic devices has resulted in ground-breaking sensing technologies. Kaaliveetil et al. [111] created a microfluidic device filled with ILs that served as a compact electrochemical gas sensor, demonstrating the capability for real-time tracking of environmental pollutants. This application highlights the adaptability of ILs across different sensing modalities, such as electrochemical and optical sensors, which are crucial for in vitro diagnostics [112].

Besides their function in fluid dynamics, ILs improve the stability and performance of a range of analytical techniques. For example, ILs have been utilized as matrices in mass spectrometry, enhancing ionization efficiency and sensitivity for intricate biological samples [113,114]. This application emphasizes the ability of ILs to enhance the analysis of biomolecules, an essential component of in vitro diagnostics. Moreover, the advancement of wearable sensors that employ ILs has garnered significant attention. Chen and Wang investigated organic IL-based sensors for healthcare applications, utilizing their distinctive properties to track physiological parameters [115,116]. These sensors provide a non-invasive method for health diagnostics, marking a notable progress in personalized medicine by facilitating continuous health monitoring and prompt interventions.

Real-world applications of IL-based diagnostic systems have started to emerge, particularly in specialized niche markets. For example, commercial IL-based electrochemical sensors for environmental monitoring, such as those developed by Metrohm (Herisau, Switzerland) and DropSens (Llanera, Spain), are already available commercially, demonstrating reliable performance in detecting pollutants and hazardous chemicals (Metrohm; https://metrohm-dropsens.com/). Additionally, Sensorix GmbH (Bonn, Germany) focuses on developing and producing ionic liquid-based gas sensors, particularly for the detection of toxic gases, illustrating further commercial advancement and practical deployment of IL-based sensor technologies (Sensorix, Germany; https://sensorix.com/satellix-sensors/1-2-trans-dce/, accessed on 11 January 2026). The transition from laboratory prototypes to commercially available diagnostic systems underscore the substantial potential of IL-based technologies to revolutionize healthcare and environmental diagnostics.

The advancement of the field highlights the considerable potential of ILs to transform diagnostics and biosensing technologies, offering promising improvements in healthcare outcomes through cutting-edge analytical solutions.

## 7. Barriers to Clinical Translation

Beyond their advantages (low vapor pressure, thermal stability, and electrochemical windows), ILs face human-health and ecotoxicity constraints. While low vapor pressure reduces risks associated with inhalation exposure and atmospheric emissions, this property alone should not be interpreted as an indicator of environmental safety or “greenness.” Environmental sustainability of ILs must instead be evaluated through a combination of toxicity, biodegradability, persistence, and bioaccumulation potential, which are largely governed by cation alkyl chain length and nanoaggregate behavior, with longer-chain cations showing decreased biocompatibility and mitochondrial accumulation. These parameters recalibrate how in vitro gains translate clinically.

Design solutions include favoring short-chain cations, biodegradable/biocompatible anions, and bio-derived components (e.g., choline-based ILs), plus early tox/PK screening (biodistribution, clearance) to derisk translation. Harmonizing with REACH/TSCA data packages and transparent AI-use disclosures in documentation improves regulatory readiness and trust [21]. ILs are highly flexible materials, and their special physicochemical properties provide many advantages to various industries. These salts, known for their astounding properties, are often utilized because they remain liquid at relatively low temperatures [117], have a very low vapor pressure [118], show outstanding thermal stability [119], provide a wide electrochemical window [120] and possess adjustable characteristics [121]. The properties of these substances have been thoroughly characterized through extensive research, including decomposition temperatures [119], ionic conductivities, viscosities [122], water solubility, densities, melting points [118], and surface tension [123]. Each of these properties is significantly affected by the interactions between their cation and anion structures [9]. The distinctive properties of ILs play a significant role in their applicability within the field of biomedicine. Their tunable characteristics and the elevated thermal stability of these substances facilitate the development of task-specific ILs tailored to fulfill the exact requirements of biomedical applications, including drug delivery [26], enzyme stabilization [124], and biomolecule preservation [125]. Furthermore, their potential for biocompatibility makes them suitable candidates for pioneering therapeutic and diagnostic technologies.

ILs present considerable benefits; however, they also could present significant risks to human health and encounter obstacles that limit their wider utilization, especially within the biomedical field. The toxicity of ILs is significantly affected by their chemical structure. Cations with long alkyl chains display higher lipophilicity, which facilitates their ability to permeate cellular membranes and interfere with normal cellular functions [126]. This may result in cytotoxic effects, including oxidative stress, apoptosis, or necrosis [127]. Liu et al. [128] investigated the harmful effects of 1-octyl-3-methylimidazolium chloride ([Omim]Cl) and tetrafluoroborate ([Omim]BF_4_) on zebrafish livers. Toxicity increased with increased doses and extended exposure, with reactive oxygen species (ROS) levels reaching 20 mg/L by day 7 and 10 mg/L by day 14, causing oxidative stress, lipid peroxidation, and DNA damage. Acute toxicity resulted in LC50 values of 152.3 mg/L for [Omim]Cl and 144.0 mg/L for [Omim]BF_4_, with no anion influence on toxicity. The acute toxicity levels indicate moderate harm at higher concentrations, while low levels show minimal effects. ILs also have the potential to interact with enzymes and proteins, leading to modifications in metabolic pathways and possibly resulting in organ toxicity or inflammatory responses with long-term exposure. For example, ILs containing imidazolium cations with different alkyl chain lengths have been found to inhibit acetylcholinesterase, with effects correlating to the lipophilicity of the cation structure [129]. Furthermore, their high stability has limited their biodegradability, which further facilitates environmental accumulation [130], thereby leading to indirect human exposure through contaminated water, soil, or food chains. To overcome the toxicity issues of ILs, one of the approaches is the functionalization of ILs by modifying their chemical structure, which results in modifications to lipophilicity and water miscibility, which are associated with subsequent biodegradability and toxicity to living organisms. This can involve the addition of oxygenated groups or the use of shorter alkyl chain cations that exhibit lower lipophilicity, thus minimizing their ability to penetrate biological membranes [9,126]. Through the examination of the toxicity of currently available ionic liquids, it becomes feasible to discern the factors contributing to their toxicity. This comprehensive understanding will facilitate the strategic design of novel classes of ionic liquids that prioritize environmental safety. Toxicological assessments should also integrate long-term studies and broad biological models, thus improving comprehension of potential harms and facilitating the finding of safer ionic liquids for biomedical applications. Enhancing the toxicity profiles of ILs, particularly in applications related to drug delivery systems and diagnostics, can be achieved while ensuring the protection of human health and environmental safety.

Another major challenge in utilizing ILs in biomedical industries is the regulatory concerns, as existing frameworks do not fully address their unique characteristics. While no specific ILs are universally approved or registered as “safe” under regulatory frameworks like the European’s REACH (Registration, Evaluation, Authorization, and Restriction of Chemicals), some ILs are assessed for industrial use under this regulation when their risks are controlled. REACH requires comprehensive safety data for chemicals, including ILs, produced or imported into the EU in quantities exceeding 1 ton annually [131]. These ILs must meet strict criteria for toxicity, environmental persistence, and biodegradability. Under the Toxic Substances Control Act (TSCA) in the US, new chemicals, including ILs, are reviewed by the Environmental Protection Agency (EPA) before commercialization. Manufacturers must submit a Premanufacture Notice (PMN), detailing the chemical’s properties and safety data [132]. While TSCA does not have a framework specifically for ILs, it evaluates their risks, including persistence and toxicity. Similarly, countries like Canada and China face similar challenges due to limited toxicological and environmental data [14], complex chemical structures [133], and biodegradability issues [134]. Factually, many ILs are custom-designed because of the high number of possible cations and anions [8,12], making it difficult to generalize findings or establish broad safety profiles. Furthermore, inconsistent testing standards and regulatory discrepancies between regions slow down the global acceptance of ILs. As the use of ILs expands, it is crucial to overcome these hurdles through improved research, collaboration, and the development of specific regulatory guidelines tailored to these substances’ distinctive characteristics [135]. A more solid global regulatory framework has the potential to improve the efficiency of commercialization processes globally while ensuring safety and promoting environmental sustainability [14].

ILs are emerging as flexible molecules with great relevance in biomedical uses. Despite challenges with toxicity, biodegradability, and regulatory barriers, ILs provide innovative approaches that can improve the safety and efficacy of conventional methods [8]. However, the examples provided in the earlier topic of the review clearly demonstrate the lack of effort to fully utilize the immense potential of ILs in the biomedical industry. Furthermore, most of the cases presented here are still at the proof-of-concept stage, and no substantial initiatives have been made to transition them from the laboratory to practical applications. By implementing targeted research and technical innovation, addressing these limitations could open up possibilities for ILs to become a crucial part of various current medicines.

## 8. Medical Application Challenges and Limitations and Economical Impact

### 8.1. Challenges and Limitations

Although ILs hold significant promise in pharmaceutical and biomedical applications, various challenges impede their wider acceptance and clinical implementation.

i. Concerns Regarding Toxicity and Biocompatibility

Although ILs are frequently praised as environmentally friendly solvents, concerns about toxicity persist, especially regarding their application in drug delivery systems and biomedical implants. Certain ionic liquids, particularly those with imidazolium and pyridinium cations, have demonstrated cytotoxic effects and possible environmental toxicity [22]. To ensure biocompatibility and minimize cytotoxicity, it is essential to carefully select and modify IL structures, focusing on the optimization of cations, anions, and functional groups.

ii. Restricted Comprehension of Lasting Consequences

The long-term bioaccumulation and metabolic pathways of ILs in living systems are still not well understood. ILs can build up in tissues or disrupt cellular functions, presenting potential risks for extended medical use [136]. Thorough in vivo studies and toxicity evaluations are essential to confirm the safety of IL in long-term therapeutic uses.

iii. Consistency in Biological Settings

The stability of ILs in physiological conditions, such as pH, temperature, and enzyme activity, continues to be a concern. Enhancing IL resistance to biological degradation through structural modifications is crucial; however, these changes frequently result in higher production costs and added complexity.

iv. Scalability of Production

The scalability of IL-based systems for medical applications remains constrained by intricate and resource-demanding synthesis methods. Creating pharmaceutical-grade ILs that comply with regulatory standards for medical use presents significant challenges and high costs, especially in terms of purification and reproducibility [137].

v. Engagement with Biomolecules

ILs frequently engage with biomolecules (such as proteins and enzymes) in unforeseen manners, which may modify their activity and functionality. For instance, they might inhibit or activate enzymes [138]. The variability presents challenges in designing ILs for highly specific biomedical applications.

### 8.2. Economic Impact of Ionic Liquids in Medical Applications

i. Elevated Production Expenses

The synthesis and purification of pharmaceutical-grade ILs continue to be prohibitively expensive, hindering their widespread adoption in the medical field [139]. Their synthesis often requires multiple steps with specialized reagents and catalysts, driving up costs. Purification is another major expense—ILs must be extensively purified (to remove impurities like halides or water), which can involve energy-intensive processes such as vacuum drying or distillation. Because ILs are usually made in small batches using custom processes, they lack economies of scale. All these factors mean the cost per kilogram of IL is much higher than that of conventional solvents or chemicals, making ILs a costly choice from the outset [140].

ii. Recycling and Reusability

Although ILs are frequently promoted as recyclable solvents, recovering and reusing ILs is challenging. In theory, ILs can be recycled to offset their high price, but in practice, this is difficult and costly. After use, ILs may be contaminated with other chemicals or degraded products, especially in pharmaceutical processes. Removing contaminants or separating the IL often requires complex steps (like liquid–liquid extraction, membrane filtration, or distillation) that themselves add cost. Moreover, each recycle can reduce the IL’s purity or effectiveness. In medical applications, where purity is critical, even trace impurities are unacceptable. As a result, companies may forgo reusing ILs or spend heavily on re-purification, limiting the economic benefit of recyclability [141].

iii. Market Viability and Scalability

The current production of many ionic liquids is confined to laboratory or pilot-scale batches. This limited scalability means ILs remain expensive and supply is not readily abundant. For medical or pharmaceutical markets, manufacturers need large-scale, consistent production—something not yet fully achieved for most ILs. The lack of established, cost-efficient mass-production techniques makes it hard for ILs to compete with cheap, bulk chemicals. In turn, high prices and uncertain supply chains reduce their market viability, so significant innovation is needed in manufacturing processes (e.g., continuous flow synthesis or cheaper raw materials) to scale production and bring costs down for broader medical use [142].

iv. Costs of Regulatory Compliance

Any material used in pharmaceuticals must meet stringent safety and quality standards, and ILs are no exception. Companies must conduct extensive toxicological studies and safety testing to prove that an IL is safe or that any residues in a drug product are within acceptable limits. Complying with Good Manufacturing Practice (GMP) is also required—ILs intended for medical use might need to be produced in certified facilities with high purity, proper documentation, and quality control, all of which raise expenses. Additionally, gaining regulatory approval for a novel IL (as an excipient or active ingredient) can be time-consuming and costly, involving paperwork, validation studies, and sometimes clinical evaluations. These compliance and certification processes significantly increase the overall cost of using ILs in medical applications [143].

v. Costs Associated with Intellectual Property and Innovation

Patents and R&D investments influence IL economics. Many ionic liquids or their specific medical applications are protected by patents. This means a company wishing to use a particular IL might have to pay licensing fees or royalties to the patent holder, adding to costs. In some cases, the most effective IL for a task might be off-limits without a license, limiting competition and keeping prices high. On the other hand, developing new ILs or formulations in-house requires substantial investment in research and development—from designing and synthesizing novel ILs to testing their performance and safety. These innovation costs (lab work, trials, and patent filings) must be recouped, often leading to higher prices for IL-based products. Intellectual property constraints and the high cost of innovation can thus slow down the widespread adoption of ILs in the medical field and concentrate use in the hands of a few organizations willing to bear these costs.

vi. Environmental and Waste Management Costs

Safe disposal of ILs is costly and necessary. Although ILs are often touted as “green” solvents (due to non-volatility and low flammability), many are not benign to the environment if released. Spent ionic liquids and waste streams containing ILs must be treated as hazardous chemical waste in many cases. This can involve special neutralization processes, containment, or high-temperature incineration to break down the ILs, all of which incur extra expenses for waste management. There are also regulatory costs: companies must comply with environmental laws when disposing of ILs, which might require permits, monitoring, and liability coverage. Therefore, ensuring that ILs do not pollute the environment (if applicable) adds another layer of costs. Waste treatment, safe disposal, and potential remediation of IL contamination contribute to the environmental cost footprint of using ionic liquids in medical applications [144].

## 9. Future Research Directions

The use of ILs for biomedical applications started in the late 1990s and early 2000s, where they were used as enhancers of thermal stability for enzymes and model proteins, as antimicrobial agents, in controlled release systems, and as formulation excipients for small molecules of low water solubility. Since then, ILs’ use expanded into these fields of applications due to their physicochemical properties and characteristics that can be task-specifically designed. According to the presented data, it is evident that the main research interest regarding biomedical applications focuses on the use of bio-ILs in order to improve the water solubility of bioactive compounds and the solubility of specific drugs via API-IL synthesis or to enhance the transdermal permeability of desirable compounds improving drug delivery.

However, many studies only focus on the physicochemical characteristics and manufacturing techniques of ILs in the biomedical field, while their biological activity has been significantly less frequently investigated. Although useful, in vitro models produce only a partial understanding of the complex relationships inside biological systems [10]. Future research directions for ILs in the biomedical field should include improving their functionality, safety, and integration into clinical practice. Therefore, to tackle the gaps in understanding the intricate interactions of ILs inside our biological systems, it is important to conduct in-vivo and clinical research. This research would provide more understanding of the pharmacokinetics, biodistribution, metabolism, and long-term effects of ILs in the body. Evaluating the biological fate of ILs, particularly their possible accumulation in tissues and their interaction with cellular components, is essential for determining their safety and efficacy [145].

New directions that can be further explored include bioengineered ILs, hybrid ILs for multifunctional uses, and ILs in personalized medicine. Bioionic liquids are a subclass of ionic liquids derived from biological molecules such as amines, sugars, and organic acids [146]. This particular IL is attractive due to its origin from renewable resources and its ability to integrate seamlessly into bio-based systems. Other than that, hybrid ILs, which are a class of ILs in which the structure incorporates functional components, such nanoparticles, polymers, or organic/inorganic moieties, or the combinations of the features of two or more different types of ILs [13] can also be further studied. ILs and their tailorable properties, as well as the rich synergistic effects they exhibit when combined with polymers, nanomaterials, or other bioactive compounds, hold great promise as the next generation of smart and multifunctional materials for biomedical and biotechnological applications [8]. These tailored combinations allow the creation of responsive systems capable of controlled drug release, targeted delivery, and real-time sensing, all while maintaining biocompatibility and structural versatility [147]. Their adaptability enables the fine-tuning of properties such as solubility, permeability, and bioavailability, making them ideal candidates for applications ranging from tissue engineering scaffolds to advanced wound dressings and implantable devices [148,149]. As the demand for more efficient, patient-specific, and sustainable therapeutic solutions increases, IL-based hybrid materials are expected to play a central role in shaping the future of biomedical innovation.

Future research should prioritize translation-oriented ionic liquid design, integrating safety, regulatory, and manufacturing considerations from the outset. This includes the development of bio-inspired and biodegradable ionic liquids, systematic evaluation of pharmacokinetics and clearance, and scalable synthesis strategies compatible with regulatory requirements.

To summarize, investigating bioengineered and hybrid ILs presents encouraging prospects for a range of uses, particularly in the biomedical field. ILs are considered one of the optimum choices for developing this industry because of their adaptable qualities and capability to combine with other materials. Going forward, interdisciplinary research combining ILs with biotechnology, materials science, pharmacology, and computational modeling will be key in developing these advanced systems and translating them into clinically viable solutions.

## 10. Conclusions

**Figure 5:**
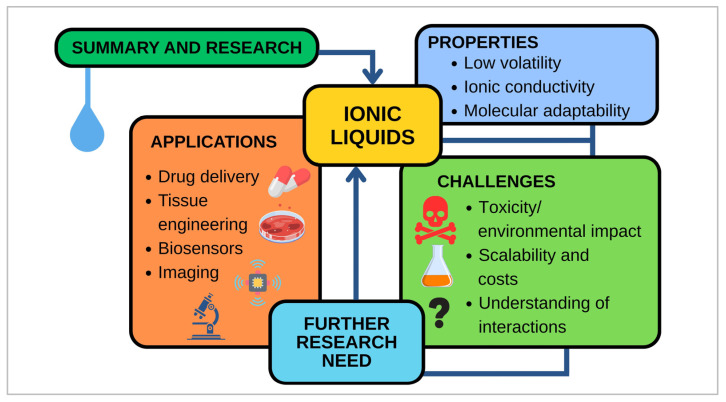
Overview of ionic liquids in biomedical applications, showcasing key properties, uses, and research challenges.

This study aims to provide a comprehensive summary and promote further research on ILs in relation to their biomedical applications. The adjustable properties of ILs, supported by the structural variety of ions and their coupling adaptability, enable them to fulfil numerous biological functions. Their unique characteristics, such as low volatility, high ionic conductivity, and molecular adaptability, have been proven to facilitate many applications including drug delivery, tissue engineering, and biosensing. Regardless of these encouraging findings, considerable research gaps and limitations persist. It is necessary to address concerns regarding toxicity and environmental impact to guarantee that ILs are safe for both human and ecological applications. The scalability IL production, along with the substantial costs linked to customized synthesis, poses significant challenges to wider adoption. Furthermore, the enduring consequences of IL exposure and their interactions within intricate biological systems necessitate additional research. The identified gaps highlight the importance for an enhanced comprehension of IL interactions within biomedical settings, as well as the advancement of sustainable and non-toxic formulations. In conclusion, ILs signify a ground-breaking advancement in various areas, especially disease treatment and diagnosis. Their versatility and adaptability position them as highly promising candidates for the development of next-generation drug delivery systems, tissue engineering scaffolds, and diagnostic platforms. Nonetheless, it is essential to confront their limitations through extensive study in order to achieve their complete potential. By addressing these challenges, ILs have the potential to revolutionize biomedical technologies, leading to safer, more effective, and environmentally sustainable solutions in the biomedical field (Figure 5).

## Figures and Tables

**Table 1:** Role and application of ILs in drug delivery systems.

Application	Cation	Anion	Drug/Component	Role of Ionic Liquids	References
Solubility	Choline ([Ch])	Glycine ([Gly]), alanine ([Al]), serine ([Ser])	Acyclovir	Enhanced solubility of acyclovir (up to 250 mg/mL in [Ch][Gly]), overcoming its poor water solubility by forming stable ionic complexes through hydrogen bonding and van der Waals interactions	[27]
	Alverinium	Tosylate, salicylate, benzoate	Alverine	Increased water solubility (e.g., tosylate IL achieves 39,937-fold solubility increase) while avoiding polymorphism	[28]
	1-ethyl-3-methylimidazolium, cholinium	Ibuprofenate	Ibuprofen	Enhanced water solubility (up to 28,000-fold compared to ibuprofen), facilitating dissolution in water and biological fluids	[29]
Bioavailability	Tetrabutylphosphonium	Edaravone	Edaravone	Improved biodistribution with longer blood circulation times and reduced renal accumulation due to nanoparticle formation	[30]
	Meglumine (MGM)	Tartaric acid, azelaic acid	Flurbiprofen	AA-MGM-IL increased flux by 3.2-fold compared to the control in transdermal delivery tests	[31]
	[C6Py], [C10Py], [C16Py], [C16MIM], [Pyc10Py]	Cefuroxime	Cefuroxime	Improved solubility–lipophilicity balance and cation-dependent modulation of octanol–water partitioning (K_o_w)	[32]
	Tetrabutylphosphonium	Gliclazide anion	Gliclazide	Improved solubility, enhanced supersaturation behavior, increased permeability, and effective incorporation into mesoporous systems	[33]
	Cholinium ([Chol)], 1-methylimidazolium ([1-MiM]), 3-picolinium ([3-Pic])	Ciprofloxacin	Ciprofloxacin	Enabled targeted delivery to resistant bacterial strains by functionalizing mesoporous silica nanoparticles with ILs. Significantly lowered minimum inhibitory concentrations for *Klebsiella pneumonia* and showed enhanced antimicrobial activity	[34]
	[LysOEt], [ValOEt], [TrpOEt]	Betulinic acid	Betulinic acid	Enhanced cytotoxicity against MCF-7 breast cancer cells. [LysOEt][BA]2 showed the highest cytotoxicity, reducing colony formation by >80% at IC_50_ concentration, with potential for selective targeting through membrane interactions	[35]

**Table 2:** Studies on IL-based biosensors.

Target Analyte	Ionic Liquid Used	Application	Reference
Prostate-Specific Antigen (PSA)	[C_4_mpyr][NTf_2_]	Cancer Biomarker Detection	[97]
Glucose, Nucleic Acids, Hormones, Biomarkers	[BMIM][PF_6_], others	Pharmaceutical Applications	[98]
Interleukin-6 (IL-6), Cortisol	[BMIM][BF_4_]	Wearable Sweat Diagnostics	[99]
Breast Cancer Biomarker CD44	[BMIM][BF_4_] + carbon nanomaterial hybrids	Electrochemical Immunosensor for Breast Cancer Detection	[95]
Alpha-fetoprotein (AFP) in saliva	[BMIM][NTf_2_] reinforced Hydroxyapatite@nano-TiO_2_	Green immuno-electrochemical sensing platform	[96]
Glucose detecttion	Imidazolium cations-ILs with Au_25_ clusters	Ionic and electronic conductivity, redox mediation	[100]
IL-6, cortisol (sweat)	Room-temperature ILs (RTILs)	Wearable non-faradaic impedimetric biosensing	[101]

## Data Availability

No new data were created or analyzed in this study. Data sharing is not applicable to this article.
